# Extracellular vesicle surface engineering with integrins (ITGAL & ITGB2) to specifically target ICAM-1-expressing endothelial cells

**DOI:** 10.1186/s12951-025-03125-3

**Published:** 2025-01-30

**Authors:** Markus Bergqvist, Kyong-Su Park, Nasibeh Karimi, Lijuan Yu, Cecilia Lässer, Jan Lötvall

**Affiliations:** Krefting Research Centre, Department of Internal Medicine and Clinical Nutrition, Institute of Medicine at Sahlgrenska Academy, University of Gothenburg, Gothenburg, Sweden

**Keywords:** Exosomes, Genetic engineering, Integrin aL, Integrin b2, Targeting, Peptide loading, Inflammation

## Abstract

Extracellular vesicles (EVs) are taken up by most cells, however specific or preferential cell targeting remains a hurdle. This study aims to develop an EV that targets cells involved in inflammation, specifically those expressing intercellular adhesion molecule-1 (ICAM-1). To target these cells, we overexpress the ICAM-1 binding receptor “lymphocyte function-associated antigen-1” (LFA-1) in HEK293F cells, by sequential transfection of plasmids of the two LFA-1 subunits, ITGAL and ITGB2 (CD11a and CD18). The LFA-1 receptor was strongly overexpressed on the EVs released by the transfected cells. We further loaded these EVs with a therapeutic peptide, targeting myeloid differentiation primary response 88 (Myd88; EV^Myd88^), through a developed EV open-and-close procedure. Myd88 is a downstream common intracellular messenger for most TLR-receptors. EV expression of LFA-1 increases EV binding to ICAM-1-expressing cells, an effect that was dose-dependently inhibited by a specific neutralizing ICAM-1 antibody. Further, activated human endothelial cells treated with LFA-1 EV^Myd88^ had increased uptake of these EVs, resulting in dose-dependent inhibition of induced release of IL-8, presumably by targeting Myd88. We conclude that LFA-1-expressing EV^Myd88^ may be a candidate suitable for delivering therapeutic peptides in inflammatory diseases associated with TLR-activation.

**Graphical Abstract:**

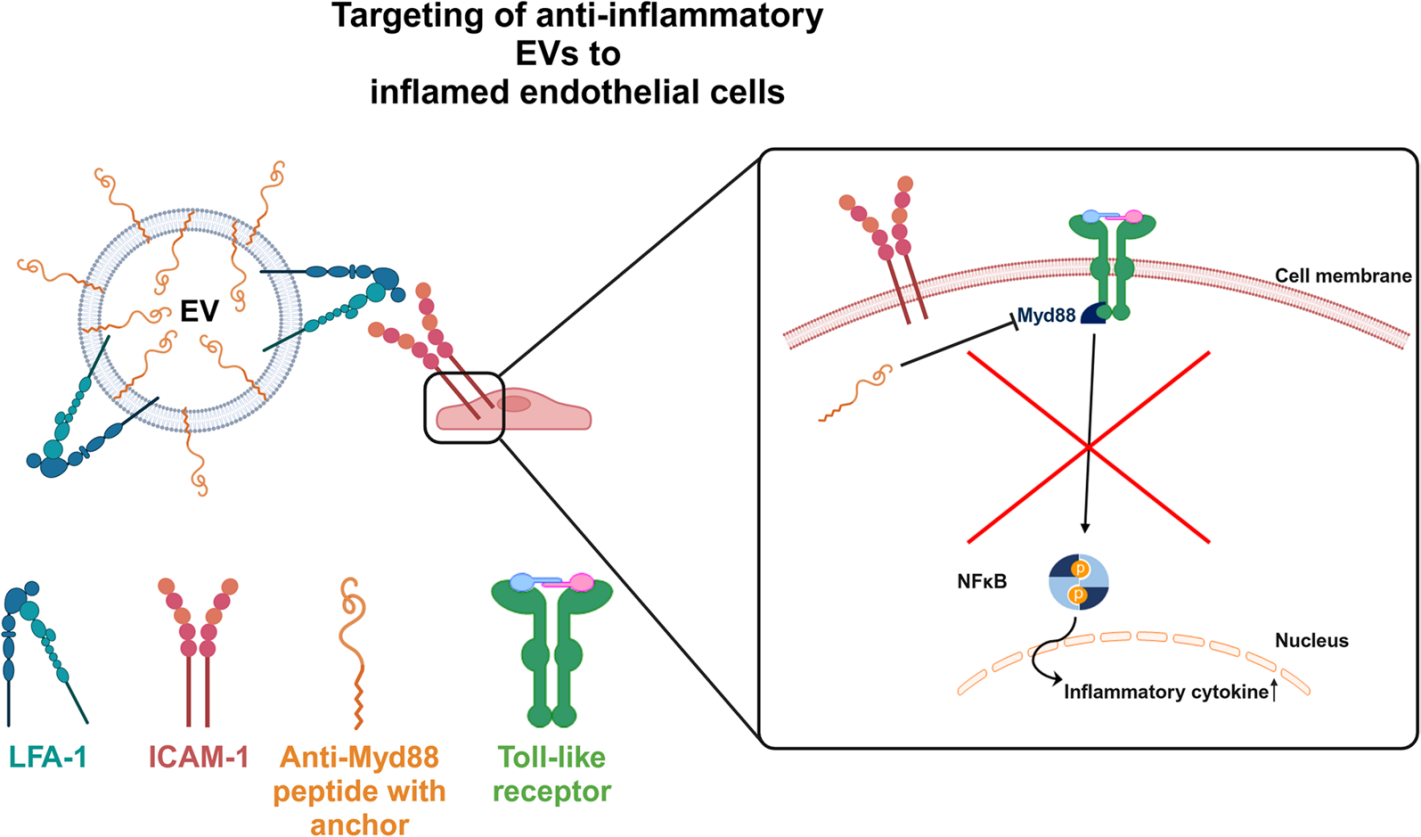

**Supplementary Information:**

The online version contains supplementary material available at 10.1186/s12951-025-03125-3.

## Introduction

Extracellular vesicles (EVs) are nanoparticles released by all eukaryotic cells, and have emerged as pivotal mediators of intercellular communication, being implicated in multiple physiological and pathological processes [1]. These small, membrane-bound vesicles are released by cells into the extracellular environment and are involved in the transfer of multiple bioactive molecules such as proteins, nucleic acids, and lipids between cells [2, 3]. The study of EVs has gained considerable attention in recent years, driven by their potential as advanced biological drugs [4]. We have previously shown that EVs can transfer functional RNA between mast cells [5], and others have shown functional protein transfer between cells by EVs [6]. The ability of EVs to shuttle molecules between different cells is expanding our understanding of the biological significance of EVs and suggests that EVs are potential drug delivery candidates to reach previously undruggable intracellular targets. Furthermore, the development of cellular engineering has broadened the opportunities in designing therapeutic EVs, both for targeting and for the delivery of therapeutic cargo [7, 8].

Different approaches can be used to improve the therapeutic and anti-inflammatory potential of EVs. First, the EVs can be engineered to be taken up by specific cells [9]. Second, they can be loaded with specific cargoes that can reach intracellular target molecules that may be involved in disease [10, 11]. Further, various strategies for loading therapeutic cargo into EVs have been developed [12]. These strategies ranged from passive loading to active engineering methods, thus highlighting the versatility of EVs for drug delivery purposes [12].

In this study, we aimed to develop EVs that target and treat inflammation. Specifically, we aimed to target intercellular adhesion molecule-1 (ICAM-1), an adhesion molecule overexpressed on multiple cells during inflammation, including vascular endothelial cells. To do this, we engineered the ICAM-1-targeting integrin lymphocyte function-associated antigen-1 (LFA-1) into EVs produced by HEK293F cells. This integrin belongs to the β2 (CD18) integrin subfamily and together with αL (CD11a) forms the functional receptor to ICAM-1 [13, 14]. LFA-1 plays a crucial role in the adhesion and trans-endothelial migration of leukocytes during immune surveillance and inflammatory responses [13]. To provide the targeting EVs with an anti-inflammatory function, EVs were loaded with an anti-inflammatory peptide targeting innate immunity responses downstream of Toll-like receptors, specifically the myeloid differentiation primary response 88 (Myd88) molecule [15]. Myd88 is a key downstream adaptor molecule for most Toll-like receptors and plays a crucial role in immune responses [16, 17]. The EVs were loaded by an open-and-close procedure, and these revesiculated EVs were generated from EVs released by engineered cells expressing LFA-1. This procedure clears EVs from cytoplasmic materials such as RNA, DNA, and cytosolic proteins, and allows intravesicular loading with smaller molecules such as the anti-Myd88 peptide (EV^Myd88^).

## Method

### Culturing cells

The HEK293F (Thermo Fisher Scientific) and HMEC-1 (ATCC-3243) cell lines were used in this study. The medium used to cultivate the HEK293F cells was Freestyle 293 medium (Thermo Fisher) with 100 µg/mL penicillin-streptomycin solution (Cytiva Hyclone). The HEK293F cells were cultured both adherently and in suspension. When cultured in suspension they were cultured in a shaking incubator with shaking at 130 rpm at 37 °C, 70% humidity, and 5% CO_2_. When cultured as adherent cultures, 10% FBS (Cytiva Hyclone) was added to the medium and the cells were cultured at 37 °C with 5% CO_2_ in a regular incubator. The HMEC-1 cell line was cultured adherently in MCDB131 medium (without l-glutamine, ThermoFisher Scientific) supplemented with 10 ng/mL human EGF recombinant protein (ThermoFisher Scientific), 1 µg/mL hydrocortisone-water soluble (Merck), 10 mM glutamine (Cytiva Hyclone), 100 µg/mL penicillin-streptomycin solution (Cytiva Hyclone), and 10% FBS (Cytiva Hyclone).

### Developing the stable LFA-1 clone

The stable LFA-1 clone was developed via sequential transfection of wild-type (WT) HEK293F cells with human CD18 and human CD11a separately. Plasmids with the sequences for CD11a and CD18 for the transfections were purchased from GenScript (Supplementary Fig. 1A and 1B). For the transfection with CD18 the cells were grown in suspension, and for the transfection with CD11a the cells were growing adherently. The transfection with CD18 of cells growing in suspension was performed using the FectoPro system (Polyplus) according to the manufacturer’s protocol. In brief, on the day of transfection the cultures were adjusted to a concentration of 2 × 10^6^ cells/mL in Freestyle 293 medium. For the transfection, IMDM media (Lonza) was used as the dilution medium. In a sterile tube, the IMDM (0.1 mL/mL final volume), plasmid (0.8 µg/mL final volume), and transfection reagent (0.8 µL/mL final volume) were combined. As a negative control, a mock transfection was performed using IMDM alone without plasmid. After a 20 min incubation at room temperature, the mixture was added to the cells and the cells were placed in a 37 °C incubator.

The transfection of adherently growing CD18 clones with CD11a was performed with the Lipofectamine 2000 system (Thermo Fisher) according to the manufacturer’s protocol. Briefly, two days prior to the transfection 1.0 × 10^6^ cells were seeded on a 6-well plate (Falcon) in 2 mL growth medium (Freestyle 293 medium with 10% FBS). On the day of transfection, the wells were roughly 90–95% confluent, and the growth medium in the wells was replaced with 1.5 mL fresh growth medium. A mixture of 4 µg of plasmid with 10 µL of the Lipofectamine 2000 transfection reagent in a total volume of 500 µL IMDM medium (Cytiva Hyclone) was allowed to incubate for 20 min at room temperature. Following this incubation, the mixture was gently mixed with the growth medium in the wells. The same mixture without the plasmid was added to the cells for the mock transfection.

### Generating a pure clone

For selection, the antibiotics G418 (500 µg/mL) and Hygro B (125 µg/mL) were added 48 h after transfection. The growth medium was changed every 48 h, and pools of clones were expanded or split as required. When the viability of the mock culture began to decline, a selection procedure was used to generate pure clones. Both the transfected and mock groups underwent identical selection processes involving seeding out the transfected cells in a series of 10× dilutions on Petri dishes. After around two weeks, 30–50 clones were picked from the Petri dishes using a pipette by gently scraping the surface of the petri dish and then collecting 50 µL of the media with the cells. The picked cells were added to a 96-well plate (Falcon) already containing 150 µL media for a total of 200 µL media per well. The clones were expanded as needed and analyzed via flow cytometry to identify the clones with the highest expression of the desired membrane proteins.

### Flow cytometry

To determine the efficiency of the transfections and the expression of the desired membrane proteins, the cells were analyzed by flow cytometry on either a BD FACS Aria II cell sorter or a BD FACSVerse flow cytometer running BD FACSSuite software (BD Biosciences). One milliliter of ice-cold FACS buffer (PBS with 1% FBS) was added to 100,000 cells, which were then pelleted at 200 × *g* for 10 min at 4 °C. The cells were then resuspended in 50 µL of human IgG (1 mg/mL in D-PBS) and incubated for 15 min at 4 °C. After this incubation, antibodies specific for the membrane proteins of interest were then added together with a viability dye for 30 min at 4 °C. The following antibodies and viability dyes were used for the staining step: BD Pharmingen FITC mouse anti-human CD18 (Clone 6.7, BD Biosciences), BD Pharmingen PE mouse anti-human CD11a (Clone HI111, BD Biosciences), BD Pharmingen 7-AAD (BD Biosciences), and Invitrogen LIVE/DEAD Fixable Aqua Dead cell stain kit (Thermo Fisher). The cells were washed with 2 mL FACS buffer and then resuspended in a final volume of 350 µL of FACS buffer. A total of 10,000 events were collected for each sample, and the data were analyzed using FlowJo software (Tree Star Inc, Ashland, OR, USA).

### Binding assay of LFA-1 cells with ICAM-1 coating

Wells in a 96-well strip plate (Thermo Fisher Scientific, Black microplate F96) were coated with ICAM-1 by introducing a 100 µL solution containing 5 µg/mL of the ligand in a coating buffer (0.1 M NaHCO_3_, pH 9.6) to the wells. Using the same concentration and procedure, wells were also coated with anti-CD18 (MEM-48, Thermo Fisher Scientific), bovine serum albumin, or human IgG as controls. Negative controls consisted of wells receiving only the coating buffer. The plates were subsequently sealed with parafilm and incubated overnight at 4 °C. Following incubation, the plates were inverted, and the solutions were removed from the wells, which were then washed twice with 150 µL of washing buffer (0.1% Tween in PBS). Subsequently, the wells were blocked with 150 µL of blocking buffer (1% BSA in PBS) per well at room temperature for 1 h, concurrent with cell labelling. Cell labelling was conducted using the fluorescent dye PKH67 (Sigma-Aldrich) following the manufacturer’s protocol. Briefly, the required number of cells was pelleted by centrifugation at 200 × *g* for 10 min, resuspended in 1 mL diluent C, and mixed with a 2× dye working solution composed of 1 mL diluent C and 4 µL PKH67. After an incubation period of 2–5 min, the reaction was halted by adding 2 mL of 1% BSA and incubating for 1 min in the dark. Excess dye was removed by washing the cells twice with PBS.

The blocking buffer was removed from the wells by inverting the plate, and the wells were washed twice with ice-cold PBS. After washing, the labelled cells were resuspended in pre-heated medium at 5 × 10^5^ cells/mL. One hundred microliters of the labelled cells (50,000 cells) were added to each well. The plate was incubated at 37 °C for 60 min to activate the receptor so that it bound to the coated ligand. After the incubation, the plates were inverted and pressed against a paper towel, and 150 µL of ice-cold PBS was then slowly pipetted into the wells and the plate was gently shaken at a low speed for three minutes. This was repeated another two times, and 100 µL of PBS was added to the wells and the plate was analyzed with a Varioskan LUX multimode microplate reader (Thermo Fisher). The wavelengths used were 480 nm for excitation and 502 nm for emission.

### EV isolation

**Fig. 1:**
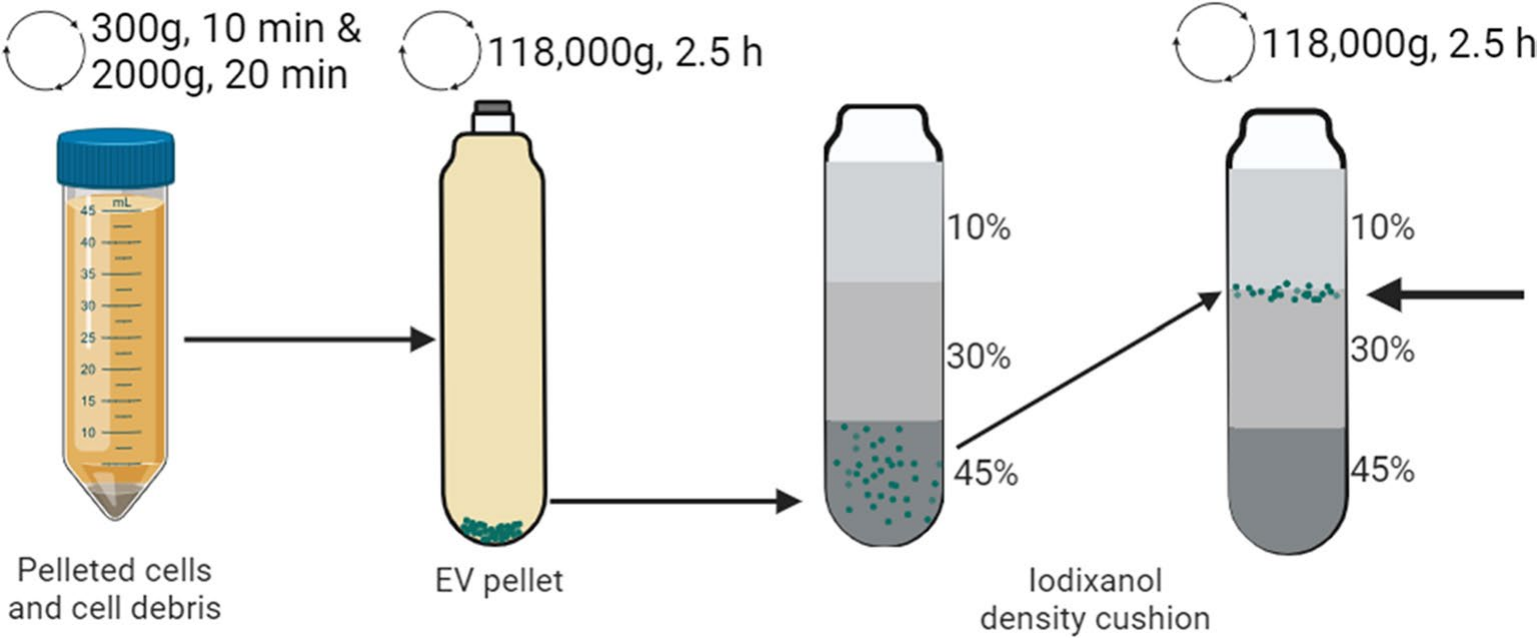
Overview of the steps for the isolation and purification of the EVs. Cells, cell debris, and large apoptotic bodies were removed by slow centrifugation (300 × *g* and 2000 × *g*). The remaining EVs were pelleted by ultracentrifugation (118,000 × *g*) and were further purified via a bottom-loaded density cushion with iodixanol (45%, 30%, and 10%)

A schematic overview of the EV isolation process is described in Fig. 1. Cells, cell debris, and large apoptotic bodies were removed from the cell culture media via centrifugation at 300 × *g* for 10 min and 2000 × *g* for 20 min. EVs were only isolated from cultures growing in suspension in FBS-free media. The supernatant was ultracentrifuged for 2.5 h at 118,000 × *g* at 4 °C (38,500 rpm, Type 45 Ti fixed angle rotor, 181 as k-factor, Beckman Coulter). The pellet was resuspended in 0.5–1 mL PBS, and the EVs were further purified via a bottom-loaded iodixanol density cushion. The cushion was generated by mixing the sample with 3 mL 60% iodixanol in the bottom of the tube (final concentration 45%) and then adding 4 mL of 30% iodixanol and 3 mL of 10% iodixanol to this layer. The sample was then ultracentrifuged at 100,000 × *g* for 2 h (28,000 rpm, SW 41 Ti swinging rotor, 265 as k-factor, Beckman Coulter). The purified EVs were collected at the 10–30% iodixanol interphase.

### EV characterization via transmission electron microscopy (TEM)

LFA-1 EVs were negatively stained and visualized by TEM. In brief, vesicles were loaded onto glow-discharged 300-mesh copper grids (Electron Microscopy Sciences) for 30 s, then washed with water twice and further stained with 2% uranyl formate for 1 min. Negative-stained EVs were analyzed by acquisition on a TALOS L 120 C transmission electronic microscope (Thermo Fisher) at 120 kV with a BM-Ceta CMOS 4k*4k CCD camera.

### Western blot

The protein concentrations of cell lysates and EVs isolated from different clones was determined with a Pierce BCA Protein assay kit (Thermo Scientific) following the recommended protocol. Samples for Western blot were made by diluting 5 µg protein from the lysates or EVs to a volume of 15 µL and then mixing it with 15 µL Sample Buffer (Bio-Rad, 2× Laemmli sample buffer), with or without dithiothreitol (DTT), to a total volume of 30 µL. DTT was used when analyzing samples for CD11a but not for CD18. Samples were added to Mini-PROTEAN TGX Stain-Free gels with seven wells (Bio-Rad) together with Precision Plus Protein Standard (Bio-Rad), using 1× Tris/Glycine/SDS Buffer (Bio-Rad). Proteins were transferred from the gel to a PVDF membrane (Bio-Rad) with Trans-Blot Turbo Mini-size Transfer Stacks (Bio-Rad) and 1× trans-blot buffer (Bio-Rad Trans-Blot Turbo 5× Transfer Buffer) using a semi-dry transfer chamber (Bio-Rad). The membrane was blocked with a blocking buffer (Bio-Rad EveryBlot Blocking Buffer) and incubated for 20 min. After this incubation, antibodies against CD11a (ab186873, Abcam, 1:1000 dilution) or CD18 (MEM-48, Thermo Fisher Scientific, 1:1000 dilution) were added and incubated overnight at 4 °C. The membrane was then washed three times with washing buffer (Bio-Rad 1× TBS with 0.1% Tween 20), incubated with the secondary antibody in blocking buffer for 60 min and then washed again three times with washing buffer. The membrane was then developed using Supersignal West Femto Maximum sensitivity substrate (Thermo Fisher Scientific) on a Chemidoc Imaging System (Bio-Rad).

### Nano-FCM of EVs

Surface molecule expression was assessed by nano-FCM using a Flow NanoAnalyzer (NanoFCM Inc.) in accordance with the manufacturer’s guidelines. Prior to sample loading, the Flow NanoAnalyzer underwent a calibration process for alignment parameters as specified by the manufacturer. This alignment involved concentration/quality control (QC beads, NanoFCM Inc.), size beads (Silica nanospheres; 68–155 and 155–850 nm; NanoFCM Inc.), and a blank control. Per the manufacturer’s instructions, the measured events per minute were maintained below 12,000 events. To achieve this, samples were initially assessed, and if the count exceeded 2000 events in the first 15 s of measurement the sample was appropriately diluted to maintain counts between 11,000 and 12,000 events.

For immunofluorescent staining, 1 µL of the prediluted antibodies at a 6-fold dilution was added to 3 µL of the diluted EV sample, followed by a 40-minute incubation at room temperature in the dark. Subsequently, samples were diluted 50 fold with PBS and promptly loaded onto the nano-FCM for data acquisition. The antibodies used for the staining step were BD Pharmingen FITC mouse anti-human CD18 (Clone 6.7, BD Biosciences) and BD Pharmingen PE mouse anti-human CD11a (Clone HI111, BD Biosciences). The instrument parameters for the Flow NanoAnalyzer were configured as follows: laser power of 10 mW at 488 nm; laser power of 20 mW at 638 nm; SS decay at 10%; sampling pressure at 1.5 kPa; time to record at 1 min; and a 525/40 filter for FITC and a 488/30 filter for PE. The nano-FCM software (NF profession V1.0) was used to calculate the percentage of positive signal, particle concentration, and size distribution.

### Proteomics—sample preparation

The amount of each sample for the proteomic analysis was normalized based on the protein content. Each sample contained 40 µg protein (9 samples), and a reference pool was also constructed with contributions from all samples containing 40 µg in total (4.44 µg/sample). Sodium dodecyl sulfate (SDS) was added to all samples to a final concentration of 2%.

The samples and reference pool were processed using a modified filter-aided sample preparation method [18]. In short, samples were reduced with 100 mM DTT at 60 °C for 30 min, transferred to Microcon-30 kDa Centrifugal Filter Units (Merck), and washed several times with 8 M urea and once with digestion buffer (DB; 50 mM TEAB and 0.5% sodium deoxycholate (SDC)) prior to alkylation with 10 mM methyl methanethiosulfonate in DB for 30 min at room temperature. Samples were digested with trypsin (Pierce MS-grade trypsin, Thermo Fisher Scientific, 1:100 ratio) at 37 °C overnight, and an additional portion of trypsin was added and incubated for another 2 h. Peptides were collected by centrifugation and labelled using tandem mass tag (TMT) 11-plex isobaric mass tagging reagents (Thermo Fisher Scientific) according to the manufacturer’s instructions. The samples were combined into one TMT-set and SDC was removed by acidification with 10% TFA. The TMT-set was further purified using a High Protein and Peptide Recovery Detergent Removal Spin Column and Pierce peptide desalting spin columns (both from Thermo Fischer Scientific) according to the manufacturer’s instructions prior to basic reversed-phase chromatography fractionation. Peptide separation was performed using a Dionex Ultimate 3000 UPLC system (Thermo Fischer Scientific) and a reversed-phase XBridge BEH C18 column (3.5 μm, 3.0 × 150 mm, Waters Corporation) with a gradient from 3 to 100% acetonitrile in 10 mM ammonium formate at pH 10.00 over 23 min at a flow of 400 µL/min. The 40 fractions were concatenated into 18 fractions, dried, and reconstituted in 3% acetonitrile and 0.1% trifluoroacetic acid.

### Proteomics—nanoLC-MS/MS analysis and data base search

Each fraction was analyzed on an Orbitrap Lumos Tribrid mass spectrometer equipped with the FAIMS Pro ion mobility system interfaced with an nLC 1200 liquid chromatography system (all from Thermo Fisher Scientific). Peptides were trapped on an Acclaim Pepmap 100 C18 trap column (100 μm × 2 cm, particle size 5 μm, Thermo Fischer Scientific) and separated on an in-house-constructed analytical column (350 × 0.075 mm I.D.) packed with 3 μm Reprosil-Pur C18-AQ particles (Dr. Maisch, Germany) using a gradient from 3 to 80% acetonitrile in 0.2% formic acid over 85 min at a flow of 300 nL/min. Precursor ion mass spectra were acquired at 120,000 resolution, scan range 375–1375, and maximum injection time 50 ms. MS2 analysis was performed in a data-dependent mode, where the most intense doubly or multiply charged precursors were isolated in the quadrupole with a 0.7 m/z isolation window and dynamic exclusion within 10 ppm for 45 s. The isolated precursors were fragmented by collision-induced dissociation at 35% collision energy with a maximum injection time of 50 ms for 3 s (‘top speed’ setting) and detected in the ion trap, followed by multinotch (simultaneous) isolation of the top 10 MS2 fragment ions within the m/z range 400–1400, and further fragmentation (MS3) by higher-energy collision dissociation (HCD) at 65% collision energy and detection in the Orbitrap at 50,000 resolution, m/z range 100–500, and a maximum injection time of 105 ms.

The data files for each set were merged for identification and relative quantification using Proteome Discoverer version 2.4 (Thermo Fisher Scientific). The search was against *Homo sapiens* (Swissprot Database, April 2023, 20,422 entries) using Sequest as a search engine with precursor mass tolerance of 5 ppm and fragment mass tolerance of 0.6 Da and Sequest HTXCorr set to 2. Tryptic peptides were accepted with one missed cleavage, variable modifications of methionine oxidation, and fixed cysteine alkylation, and TMT-label modifications of the N-terminus and lysine were selected. Percolator was used for PSM validation with the strict FDR threshold of 1%. TMT reporter ions were identified with 3 mmu mass tolerance in the MS3 HCD spectra, and the TMT reporter abundance values for each sample were normalized to the total peptide amount. Only the quantitative results for the unique peptide sequences with a minimum SPS match of 65% and an average S/N above 10 were considered for the protein quantification. The reference samples were used as the denominator and for the calculation of the ratios. The quantified proteins were filtered at 1% FDR and grouped by sharing the same sequences to minimize redundancy.

### Binding assay of LFA-1-expressing EVs to ICAM-1 cells

A binding assay was utilized to determine whether the membrane proteins CD11a and CD18 present on the EVs form a functional receptor. Wells in a 96-well plate (Thermo Scientific, Black microplate F96) were coated with EVs from the LFA-1 clone (LFA-1 EVs) by adding 10 µg/mL of the EVs in 100 µL PBS. Alternatively, wells were coated with EVs isolated from the CD18 clone (CD18 EVs) or EVs isolated from WT HEK293F cells (WT EVs), and LFA-1 EVs that had been pre-incubated with 100 µg/mL anti LFA-1 neutralizing antibody (InVivoMAb, Clone TS-1/22.1.1.13). PBS alone was added to the wells as negative controls. In these binding assays, labelled ICAM-1-expressing HMEC-1 cells were added to the wells. The HMEC-1 cells were treated with 15 ng/mL human tumor necrosis factor alpha (TNF-α; 210-TA-005, R&D systems) overnight to increase ICAM-1 expression on the cells. The washing and blocking of the plates and labelling of the cells were the same as the previous binding assay. After the labelled cells were added to the wells, the plate was incubated for 30 min at 37 °C and then washed and analyzed as for the previous binding assay.

### Uptake experiment

100,000 HMEC-1 cells were seeded in a 24-well plate (Falcon) (0.5 mL). After the cells had been allowed to adhere (3–4 h), they were treated with 15 ng/mL TNF-α (210-TA-005, R&D systems) and incubated overnight. EVs were stained with the fluorescent dye DiO by incubation for 30 min at 37 °C. The next day the cells were washed with PBS and then incubated with an anti ICAM-1 neutralizing antibody (R&D Systems, clone BBIG-I1; using concentrations 0.3﻿–30 µg/mL) or media for 90 min at 37 °C. Following this incubation the cells were again washed and incubated with either 5 × 10^9^/mL DiO-stained EVs or 5 × 10^9^/mL revesiculated EVs loaded with the fluorescent peptide and incubated for 30 min in the incubator. The wells were then washed twice with PBS and treated with 0.2 mL trypsin (Cytiva Hyclone) for 5 min at 37 °C. The trypsinated cells were then transferred to 15 ml Falcon tubes, diluted in 5 mL culture medium, and pelleted via centrifugation at 300 × *g* for 7 min. Following the centrifugation, the supernatant was removed, and the cells were resuspended in 500 µL fixation buffer (BD CellFIX) and incubated for 15 min at 4 °C. The fixed cells were washed using 1.5 mL FACS buffer (PBS with 1% FBS) followed by centrifugation at 200 × *g* for 10 min. After the centrifugation the supernatant was removed, and the cells were resuspended in 500 µL FACS buffer and analyzed via flow cytometry in which 10,000 data points were collected. The data were analyzed with FlowJo Software (Tree Star Inc.).

### Generating and loading EVs via the open-and-close procedure

To improve the anti-inflammatory potential of the LFA-1 EVs, they were loaded with the anti-Myd88 anti-inflammatory peptide via an open-and-close procedure to generate revesiculated EVs as previously described [15]. Anti-inflammatory peptides targeting Myd88 were synthetized by JPT Peptide Technologies. Attached to the Cys side chain was a fluorescent tag (Fluorescein), and the peptide sequence was Arg-Asp-Val-Leu-Pro-Gly-Tr-Cys-Val-Asn-Ser-cholesterol. This cholesterol tag allows the peptide to associate with the EVs strongly.

The LFA-1 EVs (10^11^ EVs) were treated with a high pH solution and incubated at room temperature for 30 min, after which the membrane sheets were washed with PBS. After the washing, the membranes were resuspended in 400 µL PBS with 50 µg of the peptide. The membranes were incubated with the peptides at 37 °C for 30 min after which they were sonicated for 30 min at room temperature. Peptide-loaded revesiculated EVs were separated from non-loaded peptides through iodixanol-based ultracentrifugation, using a gradient consisting of 3 mL of 60% iodixanol mixed with the sample, 4 mL of 30% iodixanol, and 3 mL of 10% iodixanol, at 100,000 × *g* for 2 h (28,000 rpm, SW 41 Ti swing rotor, 265 as k-factor, Beckman Coulter). The vesicles located in the interphase between the 10% and 30% iodixanol layers were collected. A multimode microplate reader (Varioskan LUX, Thermo Fisher Scientific) was used to determine the peptide loading efficiency with excitation and emission wavelengths set at 495 nm and 520 nm, respectively. The quantification of peptides was determined based on a calibration curve, enabling accurate calculation of the peptide loading efficiency in the collected vesicles.

### Evaluating the anti-inflammatory potential of LFA-1 EV^Myd88^

An assay was developed to determine how efficient LFA-1 EV^Myd88^ was at reducing inflammation in ICAM-1-expressing cells. HMEC-1 cells expressing ICAM-1 were seeded out onto a 24-well plate (Falcon; 100,000 cells per well in 500 µL medium) and incubated overnight. The culture medium was replaced with 500 µL fresh medium along with TNF-α (3 ng/mL; 210-TA-005, R&D systems) to induce high ICAM-1 expression and inflammatory responses in the HMEC-1 cells. The pre-treatment with TNF-α was 3 h in the incubator after which the cells were washed with PBS once and then treated with either WT EV^Myd88^ or LFA-1 EV^Myd88^ (2 × 10^9^/mL; 10,000 EVs per cell), with anti-Myd88 peptide as the positive control (2 µg; corresponding amount of peptide which was loaded in EVs) or PBS and not loaded LFA-1 EVs (2 × 10^9^/mL; 10,000 EVs per cell) as negative controls in 500 µL fresh medium for 30 min. Following this treatment, the cells were again washed with PBS and 500 µL fresh medium was added to the wells along with bacterial outer membrane vesicles (OMVs; 3 ng/mL) as a post-treatment to induce strong inflammatory responses. The OMVs were isolated as previously described using *Escherichia coli* strain DH5α [15]. The post-treatment lasted 6 h, and then the supernatant was collected and the concentration of the cytokine IL-8 was measured using a DuoSet ELISA Development kit (R&D Systems, Minneapolis, MN).

## Results

### Double transfection of HEK293F cells to express functional LFA-1

**Fig. 2:**
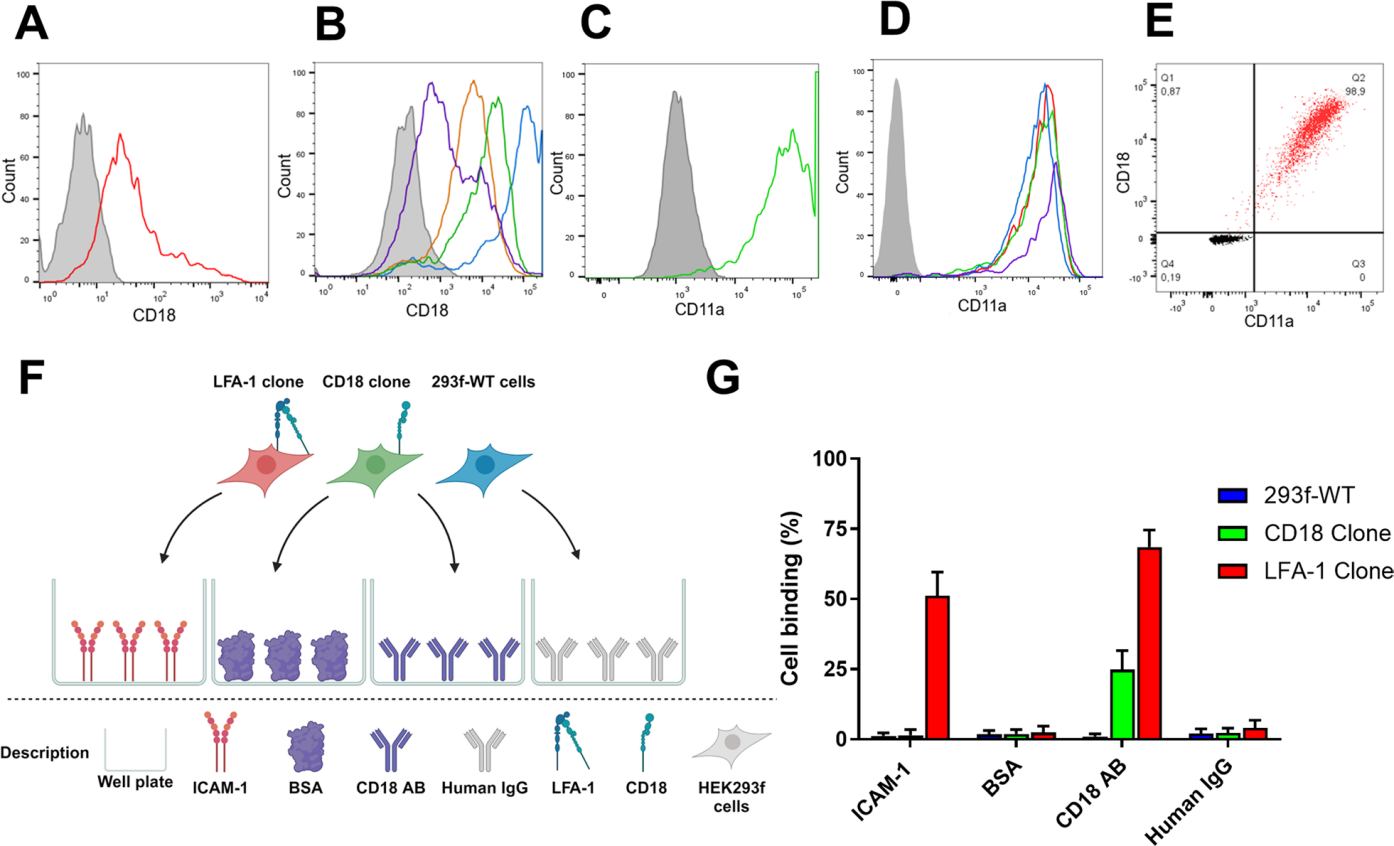
Expression of CD18 and CD11a on cell clones transfected with the respective plasmid. **A** CD18 expression of a pool of cells transfected with CD18 plasmid (red). **B** CD18 expression in four selected clones of the CD18-transfected cells from A (purple: clone #39, orange: clone #5, green: clone #29, blue: clone #3). **C** CD11a expression in CD18-expressing cells from clone #29 transfected with CD11a (green). **D** CD11a expression in four different selected clones from the CD18 clone in C transfected with CD11a. The clone with the highest expression of CD11a was LFA-1 clone #29:7 (red). **E** FACS showing co-expression of CD18 and CD11a in a purified LFA-1 clone #29:7:4 (red) and an isotype control (black). **F** Schematic overview of the binding assay used to evaluate the functionality of the receptor expressed on the LFA-1 clone. **G** Binding of the cell clones to plates coated with either ICAM-1, BSA, CD18 antibody, or human IgG isotype control. The clones of cells expressing both CD18 and CD11a efficiently bound to either ICAM-1 or anti-CD18, whereas the CD18 expressing clone (green) did not bind to ICAM-1 but bound weakly to anti-CD18 antibody-coated wells. All wells were done in duplicate in three separate experiments

A stable HEK293F clone that overexpressed CD18 and CD11a, the two membrane subunits of LFA-1, was established via sequential transfection of the two membrane subunits. CD18 was introduced to the cells via transfection of cells growing in suspension, and four CD18-positive clones were generated using the selection method (Fig. 2A and B). The clone with high expression of CD18 provided an appropriate degree of proliferation (CD18 clone #29), and was then transfected with CD11a through an adherent transfection (Fig. 2C). The pool of cells generated from the CD11a transfection underwent the same procedure as the CD18 clones. All clones picked were positive for CD11a, and the four with the highest expression are shown in Fig. 2D. The clone with the highest proliferation and expression of both CD18 and CD11a (LFA-1 clone #29:7) was re-cloned to ensure the establishment of a pure and stable cell clone. This process generated the final LFA-1 clone (LFA-1 clone 29:7:4), which was used in subsequent experiments with a clear double-positive (CD18 and CD11a) cell population of 98.9% (Fig. 2E).

The final LFA-1 clone exhibited high expression of both CD18 and CD11a. To verify that the membrane proteins formed the LFA-1 receptor once they were expressed, and that the receptor was functional, binding assays were performed using wells coated with ICAM-1 or anti-CD18 antibodies (Fig. 2F). The LFA-1-expressing cells were able to bind to and remain in the wells coated with ICAM-1, while cells expressing only CD18, and the WT HEK293F cells could not. Both the LFA-1 clone and the CD18 clone could bind to the anti-CD18 antibody, resulting in some cells remaining in the wells compared to the WT HEK293F cells. Approximately three times more LFA-1-expressing cells remained in the anti-CD18 antibody containing wells compared to the CD18-expressing cells. Few cells bound to the bovine serum albumin (BSA)-only wells, or human isotype control IgG coated wells, indicating that the ICAM-1 and anti-CD18 antibody binding was specific.

### EVs expressing LFA-1

**Fig. 3:**
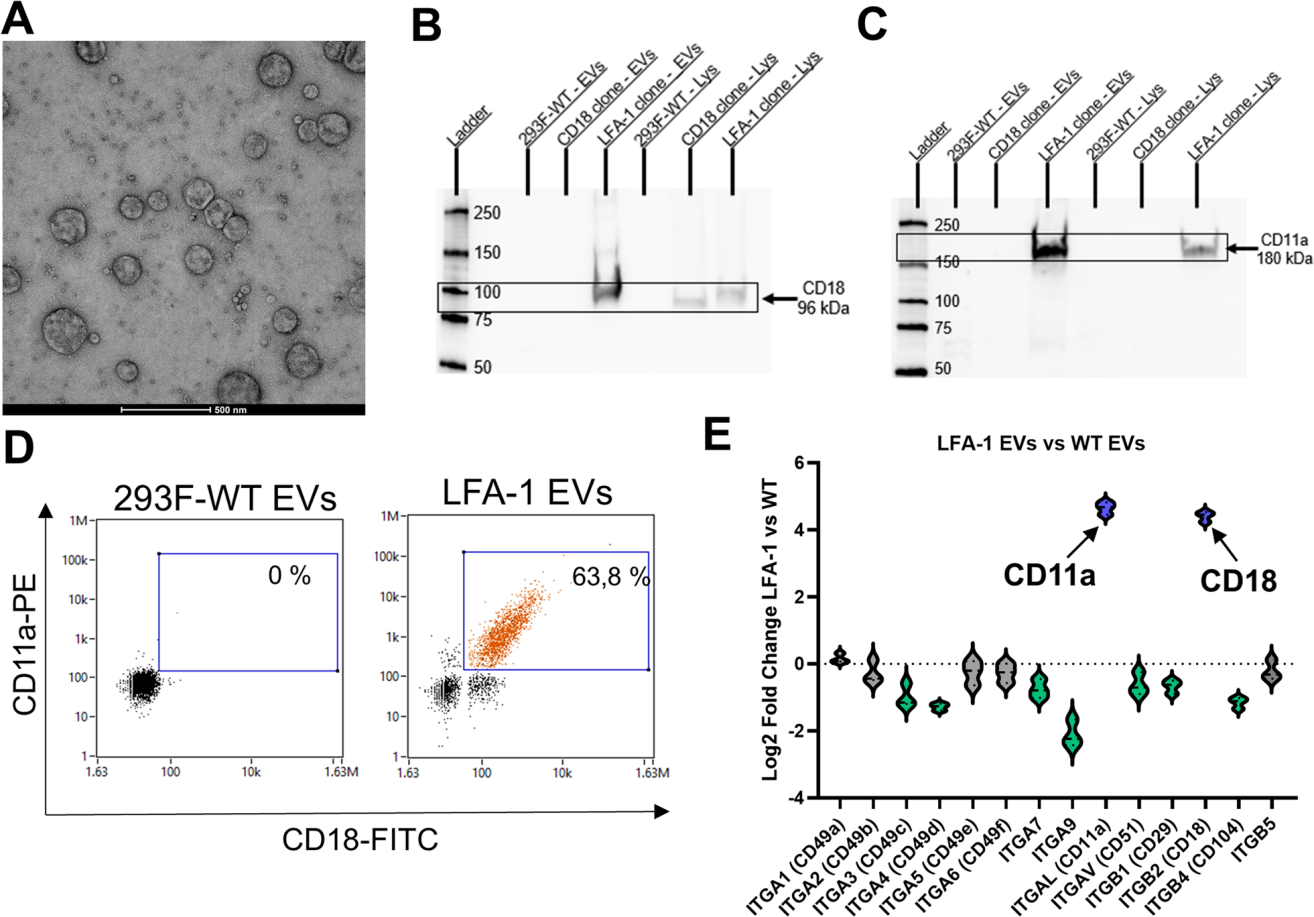
Characterization of EVs isolated from the different HEK293F clones expressing CD18 and CD11a compared to WT HEK293F cells. **A** TEM of the isolated LFA-1 EVs. **B** Western blot showing the expression of CD18 in cell lysates (Lys) and EVs isolated from WT cells, the CD18 clone, and the LFA-1 clone. **C** Western blot showing the expression of CD11a in cell lysates (Lys) and EVs isolated from WT cells, the CD18 clone, and the LFA-1 clone. **D** Nano-FCM analysis showing the expression of CD11a and CD18 in EVs isolated from WT cells, and the LFA-1 clone. **E** Quantitative proteomics analysis of the integrins present in EVs isolated from the LFA-1-expressing clone (blue) compared with EVs isolated from WT cells (green; *N* = 3)

EVs isolated from the LFA-1 clone were characterized by transmission electron microscopy (TEM; Fig. 3A), showing typical EV morphology. The maintained expression of LFA-1 on the EVs isolated from the LFA-1 clone was first confirmed with Western blot and nano-flow cytometry (nano-FCM; Fig. 3B and D). Western blot demonstrated high expression of both CD18 and CD11a in the LFA-1 EVs compared to both the lysates and the EVs from the CD18 clone and WT HEK293F cells. Interestingly, there was no band for CD18 when analyzing EV proteins from the CD18 clone, suggesting that this integrin subunit does not translocate to the EVs in high amount when expressed in the absence of CD11a. Moreover, the nano-FCM data showed that approximately 60–70% of the EVs produced by the LFA-1 clone were double positive for both CD18 and CD11a. This analysis was compared to EVs isolated from WT HEK293F cells, which were negative for both CD18 and CD11a. The TMT quantitative proteomics analysis revealed that the LFA-1 EVs overexpress both CD18 and CD11a compared to the WT EVs and CD18 EVs (Fig. 3E, Supplementary Fig. 2). Furthermore, the overall expression of several other integrins was reduced in the LFA-1 EVs, most notably the expression of the integrin ITGA9, suggesting competitive expression on the surface of EVs.

**Fig. 4:**
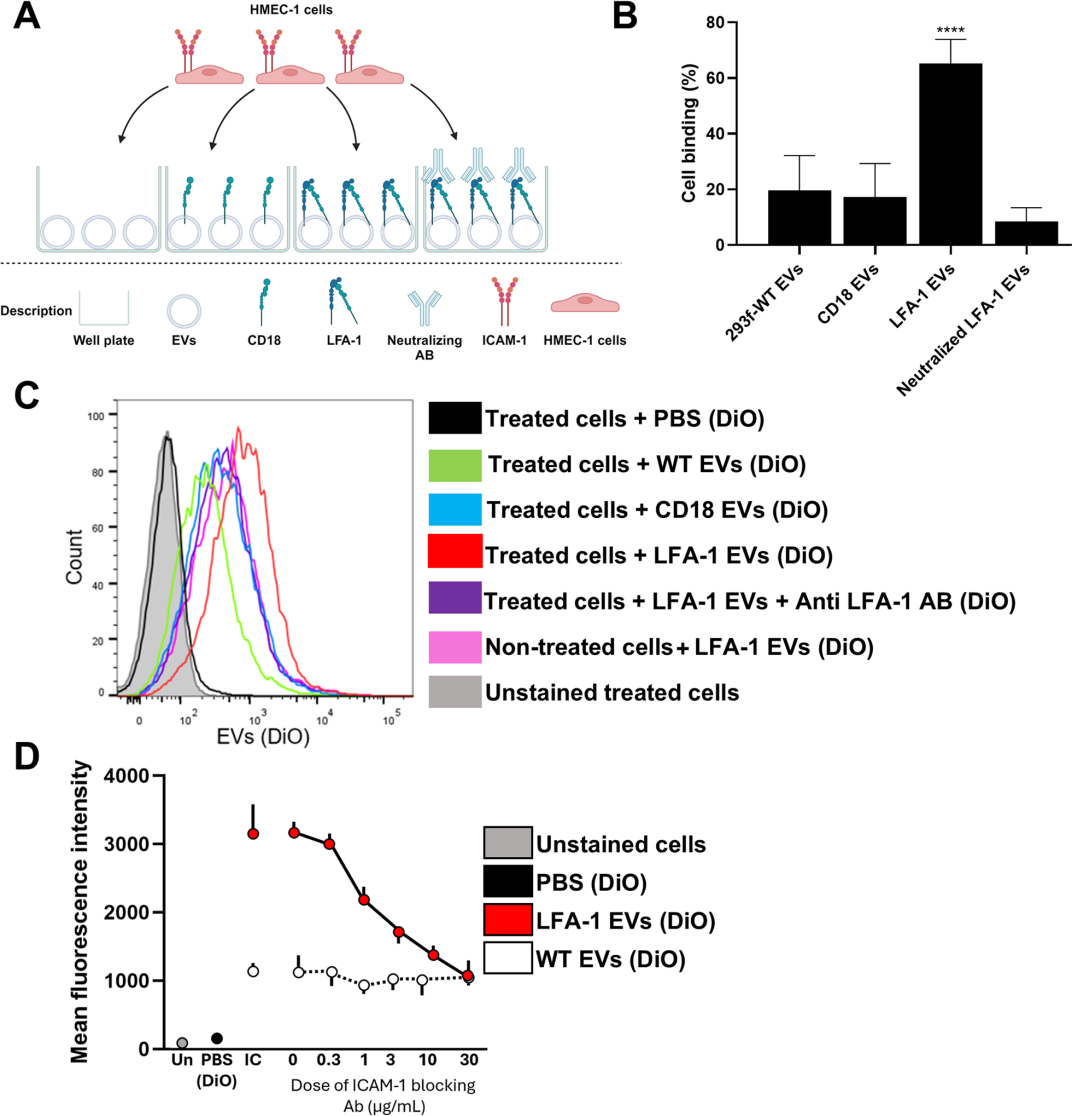
Functionality of EVs expressing LFA-1. **A** Schematic overview of the binding assay used to evaluate the functionality of the receptor expressed on the LFA-1 EVs. **B** Binding of activated ICAM-1-expressing HMEC-1 cells to wells coated with EVs from either WT HEK293F cells, the CD18 clone, or the LFA-1 clone, and the effect of an LFA-1 neutralizing antibody. All wells were done in duplicate in three separate experiments, ****P < 0.0001 by one-way ANOVA. **C** Flow cytometry measuring uptake (DiO) of EVs from the different clones, showing clearly enhanced uptake of the EVs from the LFA-1 clone (red) by the ICAM-1-expressing HMEC-1 cells. Importantly, a neutralizing LFA-1 antibody blocked the increased uptake of EVs from the LFA-1 clone (purple). **D** Competitive uptake assay with LFA-1 EVs (red) and WT EVs (white) in the presence of different doses of an anti ICAM-1 neutralization antibody, shown as mean fluorescent intensity. No ICAM-1 blocking was used for the unstained cells (Un; grey), the DiO control with PBS (black) and isotype control (IC) for LFA-1 and WT EVs (*N* = 3)

After we had validated that both CD18 and CD11a are expressed on the EVs isolated from the LFA-1 clone, we next analyzed the functionality of the receptor on the EVs. A similar assay to the binding assay with LFA-1 cells was used, but in this case, LFA-1 EVs were utilized to coat the wells, and ICAM-1-expressing HMEC-1 cells were added to the wells to determine whether they would bind to the LFA-1 EVs (Fig. 4A). More ICAM-1-expressing cells remained in the wells coated with the LFA-1 EVs compared to wells coated with WT or CD18 EVs. This indicates that the expressed LFA-1 was functional and could bind to the ICAM-1 present on the cells. To prove that the LFA-1 receptor was responsible for the binding, an LFA-1 neutralization antibody was used and demonstrated to reduce the binding (Fig. 4B).

For uptake experiments, HMEC-1 cells were activated with TNF-α to induce high expression of ICAM-1 before being incubated with the DiO-stained EVs. To determine the uptake of the EVs, the cells were analyzed by flow cytometry, where the signal from the EVs was related to the amount of EVs that were taken up using either histogram or mean fluorescent intensity analysis (Fig. 4C, D). This analysis demonstrate that the LFA-1 on the EVs facilitated their uptake compared to the other EVs, and the negative control. The other EVs included EVs isolated from a CD18 clone and WT HEK293F cells, and the negative control included DiO with PBS alone and no EVs added. Additional controls to validate that the uptake was facilitated by the LFA-1 / ICAM-1 interaction included LFA-1 EVs incubated with an LFA-1 neutralizing antibody, cells treated with an ICAM-1 neutralizing antibody and cells NOT treated with TNF-α. The experiments with an anti ICAM-1 neutralizing antibody showed a dose-dependent inhibition of the uptake of the DiO-stained LFA-1 EVs compared to DiO-stained WT EVs (Fig. 4D). Together, these data demonstrate that the engineered cells release EVs that express LFA-1, and that the LFA-1 is functional with an efficient ability to bind to ICAM-1.

### Generating peptide-loaded EVs by an open-and-close procedure

**Fig. 5:**
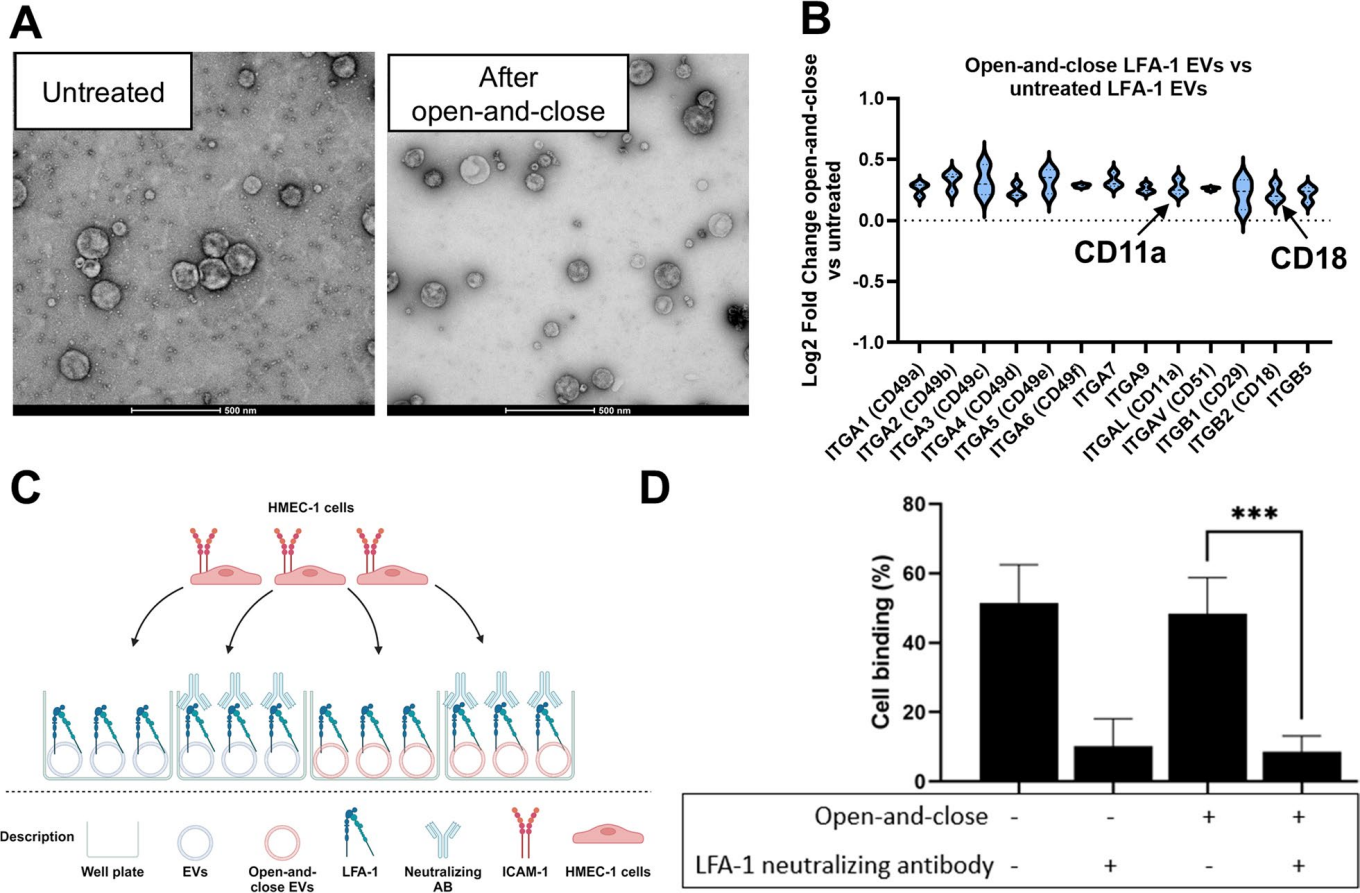
Characterization of the impact of the open-and-close procedure on LFA-1 EVs. **A** TEM images of untreated LFA-1 EVs and LFA-1 EVs after the open-and-close procedure. **B** Quantitative proteomics of the integrins present in open-and-close LFA-1 EVs compared to untreated LFA-1 EVs (*N* = 3). The relative expression of the integrins increased after the open-and-close procedure, but no differences between the expressed integrins was seen. **C** Schematic overview of the binding assay used to evaluate the functionality of the receptor expressed on the LFA-1 EVs after the open-and-close procedure. **D** Binding of activated HMEC-1 cells to wells coated with open-and-close EVs or untreated EVs expressing LFA-1. All wells were done in duplicate in three separate experiments, ***P < 0.001 by one-way ANOVA

To load the LFA-1 EVs with a potent anti-inflammatory peptide, an open-and-close procedure was performed to associate the peptide with the inside of the EV membranes. To determine that this procedure did not affect the EVs morphology and LFA-1 expression or function, TEM, quantitative proteomics and binding assays were performed. The treatment did not influence the TEM morphology of the EVs but reduced the overall background contaminants in the TEM images (Fig. 5A). The expression of integrins in the EVs was not affected by the treatment, as demonstrated by TMT quantitative proteomics analysis comparing open-and-close revesiculated EVs to non-treated EVs (Fig. 5B). If anything, the overall expression appeared to be slightly enriched, which may be attributed to the removal of cytoplasmic or contaminating proteins.

**Fig. 6:**
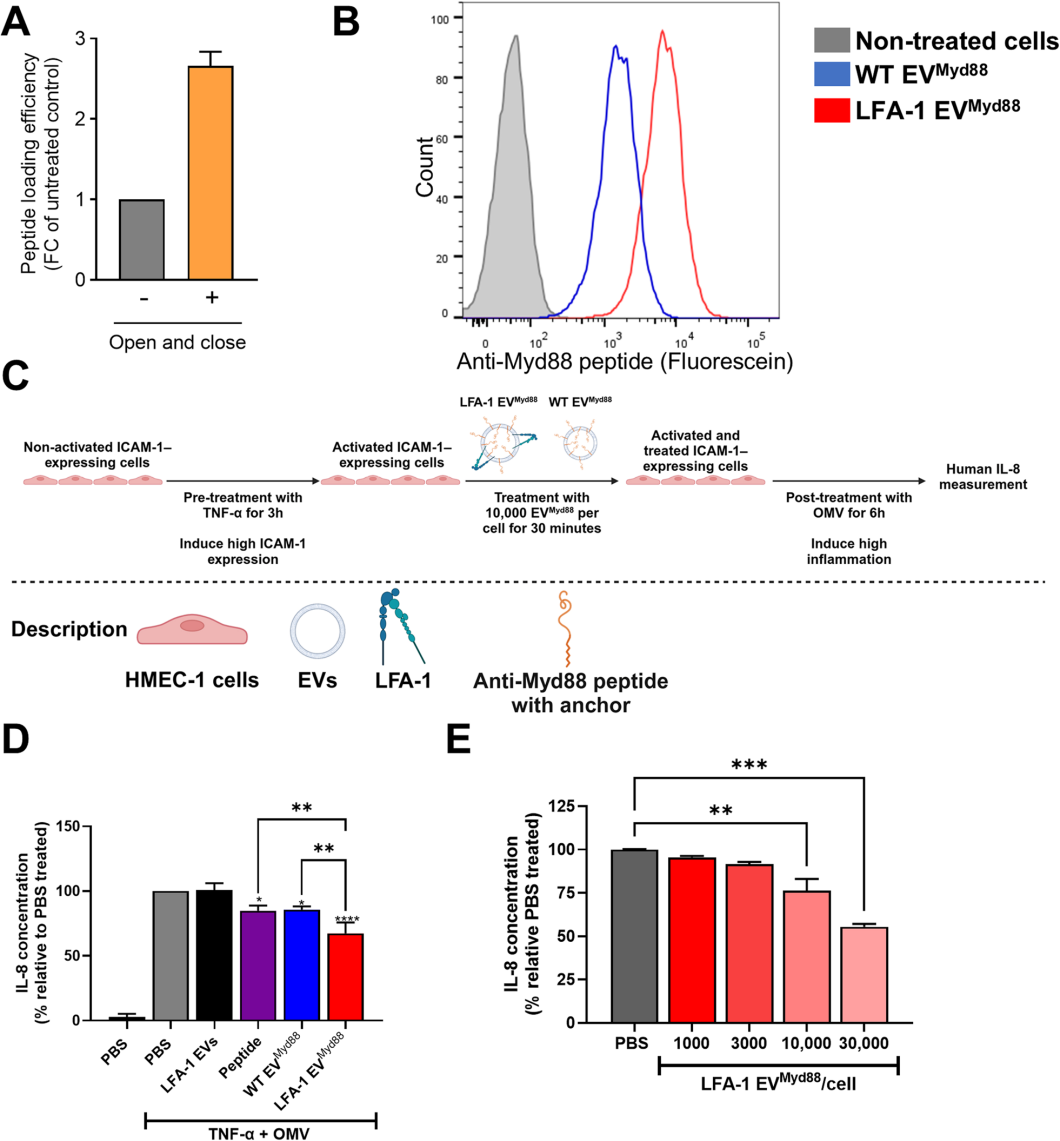
Evaluation of the loading efficiency of the open-and-close procedure and the uptake and anti-inflammatory potential of the LFA-1-expressing EVs loaded with an anti-Myd88 peptide (EV^Myd88^). **A** The loading efficiency of the open-and-close procedure compared to not doing the procedure (*N* = 3). **B** Flow cytometry measuring EV uptake into ICAM-1-expressing cells. The uptake was enhanced with LFA-1 EV^Myd88^ (red) compared to WT EV^Myd88^ (blue). Shown is one of three replicates. **C** Schematic overview of the assay used to evaluate the anti-inflammatory potential of EV^Myd88^. ICAM-1-expressing cells were briefly treated with TNF-α to induce inflammation and ICAM-1 expression in the cells. The cells were then treated with the peptide-loaded EVs. Following this treatment was a longer incubation with OMV, after which the IL-8 concentration was measured via sandwich ELISA. **D** IL-8 release after treatment of cells exposed to TNF-α and OMV. Visualized is the effect of LFA-1 EV^Myd88^ (red; 10,000 EVs per cell) compared to treating the cells with WT EV^Myd88^ (blue; 10,000 EVs per cell), not loaded LFA-1 EVs (black ; 10,000 EVs per cell) or the peptide alone (purple; 2 µg). **E** Dose-response curve for LFA-1 EV^Myd88^ using concentrations going from 1000 to 30,000 EV^Myd88^ per cell. All wells were done in duplicate in three separate experiments, **P < 0.01, ***P < 0.001 by one-way ANOVA (*N* = 3)

To exclude that the open-and-close procedure affected the functionality of the receptor, the processed EVs were used as coating in a binding assay, and were exposed to labelled ICAM-1-expressing cells (Fig. 5C). The binding capability of the LFA-1 expressed on the processed EVs was not significantly different in their ability to bind ICAM-1-expressing cells, but the binding was efficiently inhibited by an LFA-1 neutralizing antibody. During the open-and-close procedure, the loading of the vesicles with the Myd88 targeting peptide was possible, generating LFA-1 EV^Myd88^. The open-and-close procedure increased the loading efficiency by more than 2-fold compared to associating the peptide directly to the EVs without the procedure (Fig. 6A). Uptake analysis was performed by flow cytometry for the fluorescent tag on the Myd88 peptide (Fluorescein) in the recipient TNF-α-activated ICAM-1-expressing cells. The cells took up significantly more LFA-1 EV^Myd88^ if they had LFA-1 expression as compared to the WT EV^Myd88^ with no LFA-1 expression (Fig. 6B). Together this data shows that the loading of the anti-inflammatory peptide is not affecting the expression and function of the LFA-1 on EVs.

### Anti-inflammatory potential of the Myd88-peptide loaded EVs

Endothelial HMEC-1 cells were stimulated first by TNF-α (to induce ICAM-1 expression) and then by OMVs to induce high inflammation and were treated with WT or LFA-1 EV^Myd88^ (Fig. 6C). The stimulation with TNF-α and OMVs induced high ICAM-1 expression and significant IL-8 release. Treatment with LFA-1 EV^Myd88^ significantly reduced the cytokine release by more than 2-fold compared to WT EV^Myd88^ and administration of the anti-Myd88 peptide alone (Fig. 6D), showing that the expression of LFA-1 reduces the time until functional anti-inflammatory effects are observed. Furthermore, treatment of stimulated HMEC-1 cells with LFA-1 EV^Myd88^ using the same protocol reduced IL-8 release in a dose-dependent manner, with a 45% reduction observed at the highest concentration (Fig. 6E).

## Discussion

In this study, a stable HEK293F clone overexpressing the LFA-1 receptor, comprising the membrane proteins CD18 and CD11a, was successfully created via sequential transfections. These cells had increased binding to ICAM-1, a ligand for LFA-1. EVs isolated from these cells maintained high surface expression of LFA-1, and retained the increased binding to ICAM-1. After opening and emptying the EVs via ionic shock, the open vesicles were loaded with an anti-Myd88 anti-inflammatory peptide and then revesiculated, which resulted in internally loaded peptide (EV^Myd88^). Natural lipopolysaccharide (LPS)-induced cytokine release by exposure with OMVs, was significantly reduced by treating activated ICAM-1-expressing cells with LFA-1 EV^Myd88^ compared with WT EV^Myd88^ without the LFA-1 receptor. This shows that the LFA-1 expressed on EV^Myd88^ increased the uptake of the vesicles into ICAM-1-expressing cells, and the delivery of the anti-Myd88 peptide reduced cytokine release in a dose-dependent manner.

An LFA-1 expressing cell clone was developed from HEK293F cells by sequential transfection of natural human CD18 and CD11a. HEK293F cells were selected because they are easily engineered to produce and express proteins with natural functions, including post-translational modifications [19, 20]. These cells released high quantities of EVs expressing the LFA-1 integrin. Engineering of preferably HEK293F cells, but also other cell lines, to produce large quantities of EVs with different types of functional molecules has become a well-established method in the field [21, 22].

HEK293 cells have been engineered to produce EVs that carry other functional molecules on their surface, for example, IL-12 [23]. Further, HEK293 EVs can be engineered to increase loading with different soluble intravesicular molecules [24]. Often, these studies have used overexpression of membrane scaffold proteins to achieve the desired surface protein density. HEK293 cells have also been genetically engineered to produce EVs to improve their targeting capabilities, for example by expressing an ICAM-1 fusion protein on the EVs in order to target T cells [25]. Similarly, in the current study, we overexpressed the natural transmembrane components of the human integrin LFA-1, thus avoiding the inclusion of potentially immunogenic non-self scaffold proteins [13]. The LFA-1 expression on the cells and the EVs resulted in increased binding to the ligand ICAM-1 without any additional structural change of the LFA-1 integrin, even though previous studies have suggested that cytokines and cations are required to make LFA-1 functional [26–28].

After isolating EVs from the different clones and the WT HEK293F cells, western blot analysis showed that EVs from cells engineered to express CD18 without CD11a were negative for CD18, even though the source cells were positive for membrane-bound CD18. However, co-expression of CD18 with CD11a in the cells resulted in the production of EVs with the expression of both CD18 and CD11a. Interestingly, the overexpression of LFA-1 resulted in a reduction in the expression of some other integrins in EVs, notably ITGA9. This suggests that there is a functional or competitive regulation of protein expression on EVs, which indeed has been indicated previously [29]. That study suggested that overexpressing the desired proteins in cells, and subsequently in EVs, can alter the composition of other surface proteins on the EVs [29]. The specific mechanisms for the integrin expression and regulation in EVs has been suggested to be similar to that in cells, but this remains to be confirmed [28, 30].

The expression of LFA-1 on the EV membrane resulted in increased specific binding to ICAM-1, which could be blocked by an LFA-1 neutralizing antibody. Furthermore, the LFA-1 expression on the EVs resulted in increased uptake into human endothelial cells expressing ICAM-1, which also could be blocked by a neutralizing LFA-1 or ICAM-1 antibody. Although the DiO-stained EVs and EV^Myd88^ were not visualized to enter the inside of recipient ICAM-1-expressing cells, the Myd88 targeting peptide was found to be functional, which would only happen intracellularly. We have previously utilized adhesion molecules on cell-derived vesicles to increase endothelial uptake and functionality [31]. However, at that time we utilized the natural expression of an array of multiple adhesion molecules on the inflammatory cell to produce vesicles generally homing to inflamed tissues. However, at that stage, we were unable to identify which specific adhesion molecules were potentially involved in the EV-homing to the inflamed tissue.

To load the targeting EVs with an anti-inflammatory peptide, we used an ionic shock protocol, which opens the EVs and allows loading with a Myd88-targeting peptide [15], followed by closing of the EVs. This procedure also reduces the quantity of cytosolic proteins and RNA present in the processed EVs [15]. In this study, we also showed that the procedure did not influence EV morphology or the expression of functional integrins on EVs. Furthermore, this procedure allow internal EV loading of the peptide, which significantly increases the numbers of peptides per EV (Fig. 6A), compared to passive loading when the peptide can only be externally loaded onto the vesicles using a cholesterol tag [32]. It is possible that the initial emptying of the EVs through the ionic shock treatment empties the vesicles of cytosolic molecules, giving room for more peptides in the intravesicular space. Also, TEM imaging of the ionic-shock treated and revesiculated EVs suggests that the general protein background is significantly improved (Fig. 5A). Importantly, the ionic-shock treated EVs maintain efficient uptake to ICAM-1-expressing cells vs WT EVs.

ICAM-1 has played a pivotal role in drug delivery systems due to its upregulation in various inflammatory diseases, and early strategies have involved monoclonal antibodies to block ICAM-1 to thereby reduce inflammation [33–35]. However, with advances in nanotechnology, ICAM-1-targeted delivery systems emerged, utilizing nanoparticles, liposomes, nanogels and polymers to enhance the specificity of drug delivery to inflamed or diseased tissues [36–40]. These different drug delivery systems have utilized the overexpression of ICAM-1 on endothelial cells to achieve relatively selective targeting both in vitro and in vivo, minimizing off-target effects and improving therapeutic efficacy. However, these systems target ICAM-1 using antibodies bound to the carriers compared, to the use of EVs in the current study, produced by cells engineered to overexpress LFA-1, the natural ligand of ICAM-1. To ensure high ICAM-1 expression on the endothelial cells to evaluate the anti-inflammatory potential of the EV^Myd88^, the cells were first incubated with human TNF-a before exposed to the EVs. Following the EV-treatment the cells were further incubated with OMV to induce high release of IL-8, a Myd88 dependent cytokine [15].

Peptides have the potential to treat an array of different types of diseases, which has achieved increased interest over the last decades [41]. Most famous is the application of peptides targeting the GLP pathway in diabetes and obesity [41]. However, different peptides have also been tested in models of inflammatory diseases, including inflammatory bowel disease and autoimmune encephalitis [42]. To treat these conditions, a study used a peptide called Tkip that binds and inhibits the tyrosine Janus kinase 2 in vitro [43]. Other inflammatory pathways that have been tested with experimental peptides in rheumatic arthritis models [44]. Briefly, a 15-mer peptide (SAP15) derived from the human immune-related biomolecule b-defensin 3 has been used. The SAP15 peptide targeted intracellular histone deacetylase 5 (HDAC5) and this interaction reduces LPS-induced phosphorylation of the HDAC5, and thus reduces the inflammation [44].

Targeting the Myd88 pathway using a peptide loaded into EVs (EV^Myd88^), had been described to be efficient in a model of sepsis [15], although in that study non-targeting but anti-inflammatory Mesenchymal Stem Cell vesicles were used [15], which enhanced peptide functionality in vitro. As described in the current study, EV-specific targeting to ICAM-1 can also potentiate the biological function of the loaded peptide, synergizing anti-inflammatory effects. Indeed, this study is the first to utilize such an approach to pharmacologically deliver a peptide to the intracellular space of specifically ICAM-1-expressing cells, such as endothelial cells. Targeting endothelium in severe inflammation is important to retain vascular integrity, and to reduce inflammatory transmigration to sites of inflammation [45]. This study thus suggests that utilizing EVs to deliver peptide drugs can be highly efficient, especially if the EV has a targeting capacity towards specific receptors on specific cells. Further, the peptide we have used is targeting innate immunity, which classical anti-inflammatory molecules such as glucocorticoids are inefficient to treat [46].

In conclusion, the expression of CD11a and CD18 in HEK293F cells results in their release of EVs with high expression of LFA-1 that targets the adhesion molecule ICAM-1. Further, when we process these EVs with an open-and-close procedure, we can efficiently load them with a therapeutic peptide, which in this case was selected to target the innate immunity-associated intracellular messenger Myd88. The LFA-1-expressing EVs loaded with the therapeutic peptide were rapidly taken up by ICAM-1-expressing cells, and the peptide exhibited significant anti-inflammatory effects. This study thus provides a comprehensive description of a potential therapeutic EV with specific cell-targeting capacity, and with efficient intracellular delivery of an anti-inflammatory therapeutic peptide, which can be useful in treating inflammatory processes.

## Supplementary Information

Supplementary material 1: Supplementary Fig. 1. Plasmids used for the two transfections in this study. A , The plasmid used in the transfection for the expression of CD11a with Hygromycin B as the selection marker. B , The plasmid used in the transfection for the expression of CD18 with G418 as the selection marker. Supplementary Fig. 2. Quantitative proteomics of the integrins present in EVs isolated from different clones and WT HEK293F cells (N = 3). A , The relative expression of integrins comparing EVs isolated from the LFA-1-expressing clone (blue) and the CD18-expressing clone (red). B , The relative expression of integrins comparing EVs isolated from the CD18-expressing clone (red) and WT HEK293F cells (green).

## Data Availability

The datasets used and analyzed in the current study are available upon request from the corresponding author or MB.
