# Supplementary figures and images for: Extracellular vesicle surface engineering with integrins (ITGAL & ITGB2) to specifically target ICAM-1-expressing endothelial cells
Source: J Nanobiotechnology. 2025 Jan 30;23:64. doi: 10.1186/s12951-025-03125-3 (PMC11780982; doi:10.1186/s12951-025-03125-3)

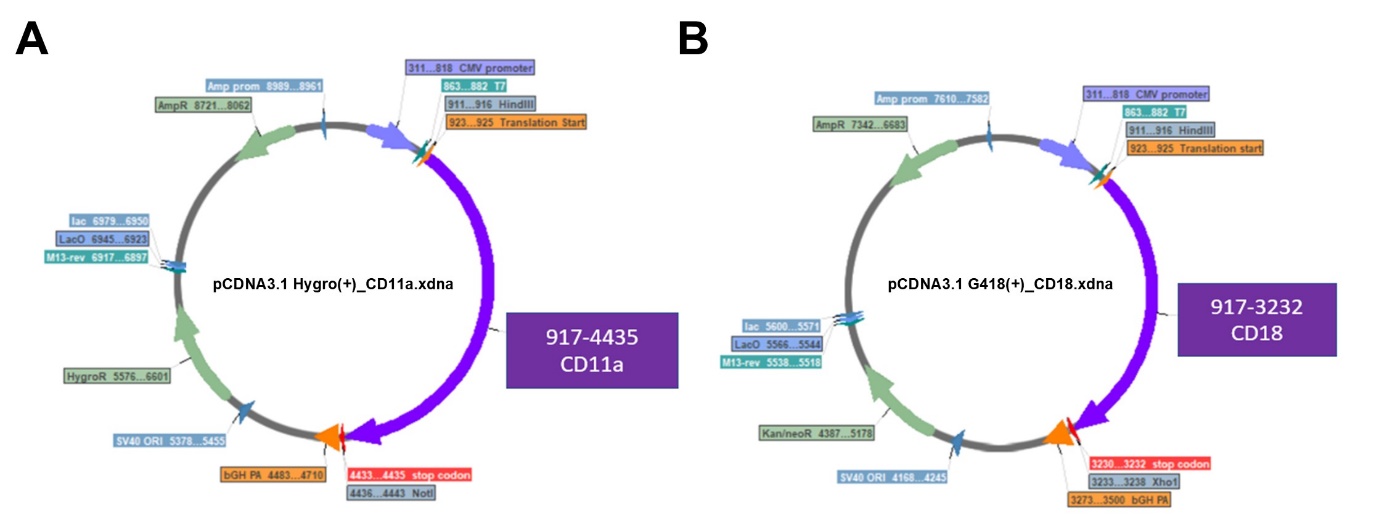


**Supplementary Figure 1**


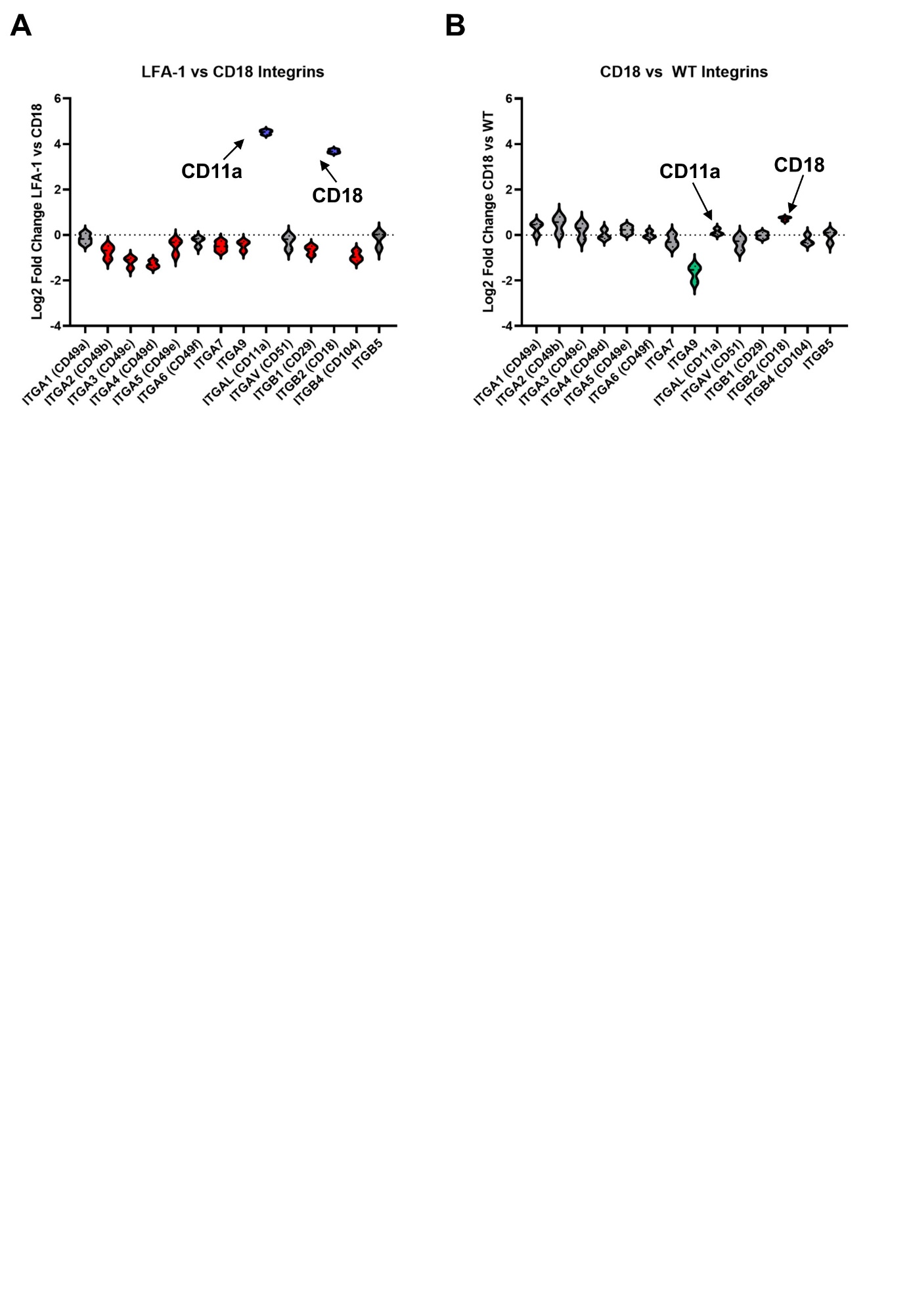


**Supplementary Figure 2**

Supplement: Supplementary file 1 — Supplementary material 1: Supplementary Fig. 1. Plasmids used for the two transfections in this study. A , The plasmid used in the transfection for the expression of CD11a with Hygromycin B as the selection marker. B , The plasmid used in the transfection for the expression of CD18 with G418 as the selection marker. Supplementary Fig. 2. Quantitative proteomics of the integrins present in EVs isolated from different clones and WT HEK293F cells (N = 3). A , The relative expression of integrins comparing EVs isolated from the LFA-1-expressing clone (blue) and the CD18-expressing clone (red). B , The relative expression of integrins comparing EVs isolated from the CD18-expressing clone (red) and WT HEK293F cells (green). [file 12951_2025_3125_MOESM1_ESM.docx]
